# Diagnostic Strategies for Breast Cancer Detection: From Image Generation to Classification Strategies Using Artificial Intelligence Algorithms

**DOI:** 10.3390/cancers14143442

**Published:** 2022-07-15

**Authors:** Jesus A. Basurto-Hurtado, Irving A. Cruz-Albarran, Manuel Toledano-Ayala, Mario Alberto Ibarra-Manzano, Luis A. Morales-Hernandez, Carlos A. Perez-Ramirez

**Affiliations:** 1 C.A. Mecatrónica, Facultad de Ingeniería, Campus San Juan del Río, Universidad Autónoma de Querétaro, Rio Moctezuma 249, San Cayetano, San Juan del Rio 76807, Mexico; jesus.alberto.basurto@uaq.mx (J.A.B.-H.); irving.cruz@uaq.mx (I.A.C.-A.); 2 Laboratorio de Dispositivos Médicos, Facultad de Ingeniería, Universidad Autónoma de Querétaro, Carretera a Chichimequillas S/N, Ejido Bolaños, Santiago de Querétaro 76140, Mexico; 3 División de Investigación y Posgrado de la Facultad de Ingeniería (DIPFI), Universidad Autónoma de Querétaro, Cerro de las Campanas S/N Las Campanas, Santiago de Querétaro 76010, Mexico; toledano@uaq.mx; 4 Laboratorio de Procesamiento Digital de Señales, Departamento de Ingeniería Electrónica, Division de Ingenierias Campus Irapuato-Salamanca (DICIS), Universidad de Guanajuato, Carretera Salamanca-Valle de Santiago KM. 3.5 + 1.8 Km., Salamanca 36885, Mexico; ibarram@ugto.mx

**Keywords:** breast cancer, mammography, magnetic resonance, ultrasound, thermography, image processing, artificial intelligence

## Abstract

**Simple Summary:**

With the recent advances in the field of artificial intelligence, it has been possible to develop robust and accurate methodologies that can deliver noticeable results in different health- related areas, where the oncology is one the hottest research areas nowadays, as it is now possible to fuse information that the images have with the patient medical records in order to offer a more accurate diagnosis. In this sense, understanding the process of how an AI-based methodology is developed can offer a helpful insight to develop such methodologies. In this review, we comprehensively guide the reader on the steps required to develop such methodology, starting from the image formation to its processing and interpretation using a wide variety of methods; further, some techniques that can be used in the next-generation diagnostic strategies are also presented. We believe this helpful insight will provide deeper comprehension to students and researchers in the related areas, of the advantages and disadvantages of every method.

**Abstract:**

Breast cancer is one the main death causes for women worldwide, as 16% of the diagnosed malignant lesions worldwide are its consequence. In this sense, it is of paramount importance to diagnose these lesions in the earliest stage possible, in order to have the highest chances of survival. While there are several works that present selected topics in this area, none of them present a complete panorama, that is, from the image generation to its interpretation. This work presents a comprehensive state-of-the-art review of the image generation and processing techniques to detect Breast Cancer, where potential candidates for the image generation and processing are presented and discussed. Novel methodologies should consider the adroit integration of artificial intelligence-concepts and the categorical data to generate modern alternatives that can have the accuracy, precision and reliability expected to mitigate the misclassifications.

## 1. Introduction

According to the World Health Organization, Breast Cancer (BC) represents around 16% of the malignant tumors diagnosed worldwide [1]. In Mexico, BC is the leading death cause for cancer in the female population [2]. BC develops when any lump begins an angiogenesis process, that is, the process that causes the development of new blood vessels and capillaries from the existent vasculature [3]. Unfortunately, BC has a mortality rate of 69% in emergent countries, which is greater than the one in developed countries [1]. This increase is explained as the cancer is detected in a later stage, making the treatment a financial obstacle as its price increases, especially if the disease is detected in an advanced stage [4]. Hence, the development of strategies that can perform an early detection of BC is a priority topic for governments, as an early detection increases the survival chances and lowers the financial burden the disease imposes to families and health systems [4].

**Figure 1:**
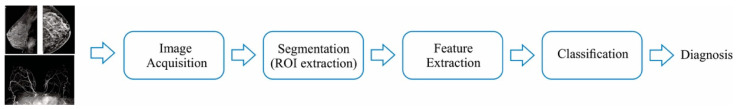
BC detection using image processing strategies.

A methodology for the BC detection can be composed of 4 steps: (1) image acquisition, (2) Segmentation and preprocessing, (3) feature extraction, and (4) classification. An illustration of the abovementioned concepts is described in Figure 1.

From this figure, it can be seen that the first step uses the different technologies available to acquire the internal tissue dynamics of the breast, so they can be expressed in an image; the second step is used to execute algorithms that perform basic tasks on the images (for instance, correcting the color scale), so the segmentation, which is the detection of Region-of-interest (ROI), can be done; then, the third step quantifies the differences between images that have abnormalities from the ones that do not have; finally, once the differences are quantified, it is necessary to classify them to provide a diagnosis. With the rapid development of novel technologies that can capture more accurately the dynamics of the breast tissues, numerous advances have been done in all the aforementioned fields; in this sense, the goal of detecting all the abnormalities without generating false alarms is still a highly desirable feature for all the proposals [5,6]. Recently, some articles have reviewed some proposals regarding the feature classification and its interpretation [6,7,8,9]; yet, an article that presents the main technologies used to form the breast image as well as the processing stages required to provide a diagnosis is still missing. This article presents a state-of-the-art review of both the technologies used to create the breast image as well as the strategies employed to perform the image processing and classification. The article is organized as follows: Section 2 describes the main technologies used for the image generation; Section 3 describes the methods used to perform the segmentation, feature extraction, as well as the interpretation; next, Section 4 and Section 5 present some emerging techniques that can be used to improve the image formation and the algorithms used for the interpretation. The article ends with some concluding remarks.

## 2. Technologies Used to Obtain Breast Tissue Images

One of the steps require to develop a diagnose system is the representation of the breast tissue dynamics. In this sense, there are several technologies that are commonly used to represent the tissue by means of images. This section presents the most used ones.

### 2.1. Mammography

**Figure 2:**
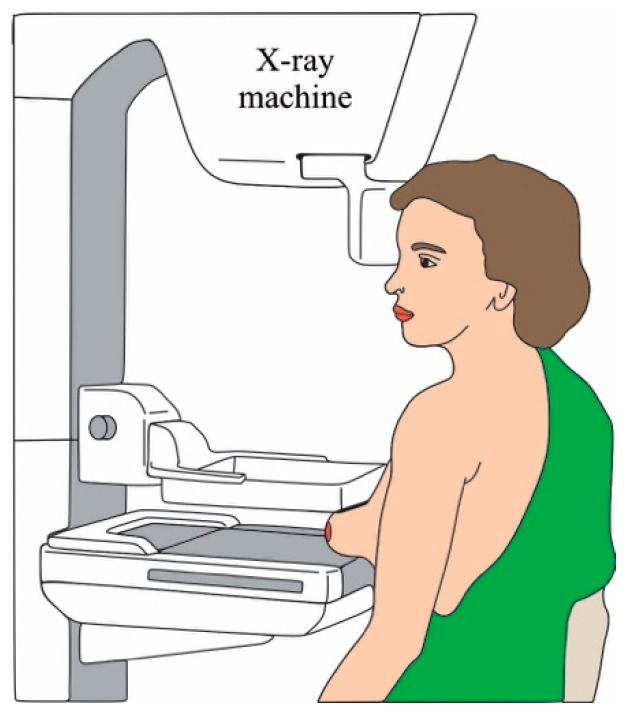
Mammography procedure.

Mammography is a study used to screen the breast tissue in order to detect abnormalities that could indicate the prescience of cancer or other breast diseases [10]. This technique has a sensibility of up to 85% in the recommended population. Essentially, mammography uses low doses of X-ray to form a picture of the breast internal tissues [11]. To form the picture, the breasts are compressed by two plates with the aim of mitigating the dispersion of the rays, allowing to obtain a better picture without using an X-ray high-dose [11], where the tissue changes might appear as white zones on a grey contrast [11]. On average, the total radiation dose for a typical mammogram with 2 views for each breast is about 0.4 [11]. Figure 2 illustrates the mammography procedure.

Several works have focused on the processing of the digital mammographies to detect the most common symptoms that could indicate the presence of cancer: calcifications or masses [12]. Traditionally, the specialist looks for zones that have a different appearance (size, shape, contrast, edges, or bright spots) than the normal tissue. With the employment of segmentation algorithms [13,14,15], the automatization of this task has been proposed, where some attempts using neural networks have done [12,16,17], delivering encouraging results.

Recently, the utilization of the Breast tomosynthesis (BT) and the Contrast-Enhanced Mammography (CEM) [10] have been proposed as improvements to the traditional digital mammography. The former is a 3D breast reconstruction that allows to further improve the image resolution whereas the latter improves the image resolution injecting a contrast agent; in this way, the anatomic and vascularity definition of the abnormalities is exposed. In this sense, some improvements when dealing with breast-dense tissue patients are obtained; yet, the detection of clustered micro calcifications is still an issue [10]; on the other hand, additional screening tests are required to determine if the abnormality detected by CEM is cancer or not, besides of requiring more expensive equipment.

### 2.2. Ultrasound

**Figure 3:**
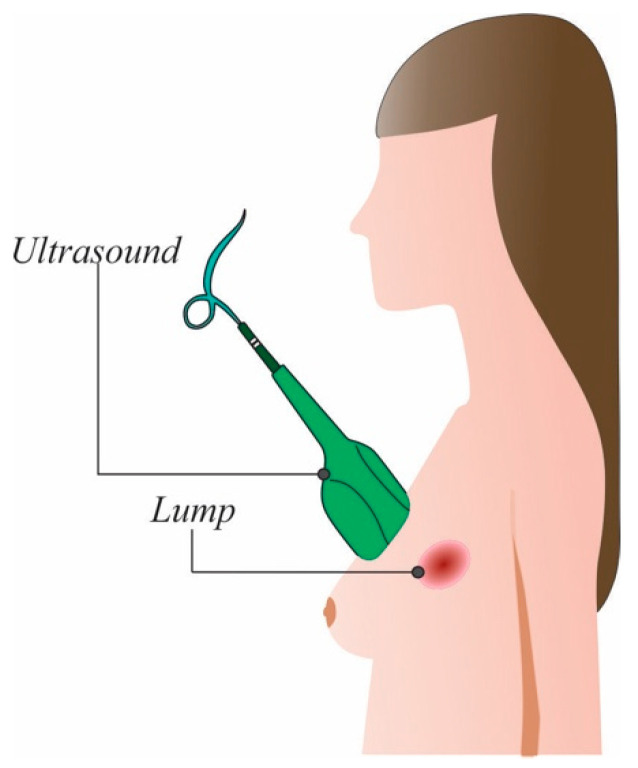
Ultrasound procedure.

Ultrasound is a non-invasive and non-irradiating technique that uses sound waves to create images from organs, in this case the breasts, to detect changes in their form. To create the images, a transducer sends high-frequency sound waves (>20 kHz) and measures the reflected ones [10]. The image is formed using the wave sound reflected from the internal tissues. This procedure is depicted in Figure 3.

Ultrasound is used for three purposes: (1) assessing and determining the abnormality condition, that is, to help doctors if the abnormal mass is solid, which might require further examination, is fluid-filled, or has both features; (2) as an auxiliary screen tool, which is used when the patient has dense breasts and the mammography is not the reliable enough, (3) or as a guide to develop a biopsy in the suspected abnormality [10]. Several computer-aided diagnose (CAD) systems that analyze ultrasound images have been proposed [18]. One of the points they note it is necessary to improve is the resolution of the images [19] using specific-designed filters. Another modification proposed is the utilization of micro-bubbles that are injected into the abnormalities detected at first sight [20].

It should be noticed that the mass tends to stay in its position when compressed, i.e., they do not displace. Elastography is the technique that is employed to measure the tumor displacement when compressed using a special transducer [21]. These developments have led to discover masses that usually require performing a biopsy to determine the mass nature, which delay the diagnosis confirmation [10,21]; moreover, the image interpretation requires a well-trained specialist, which is not always available to perform all the studies.

### 2.3. Magnetic Resonance Imagining (MRI)

**Figure 4:**
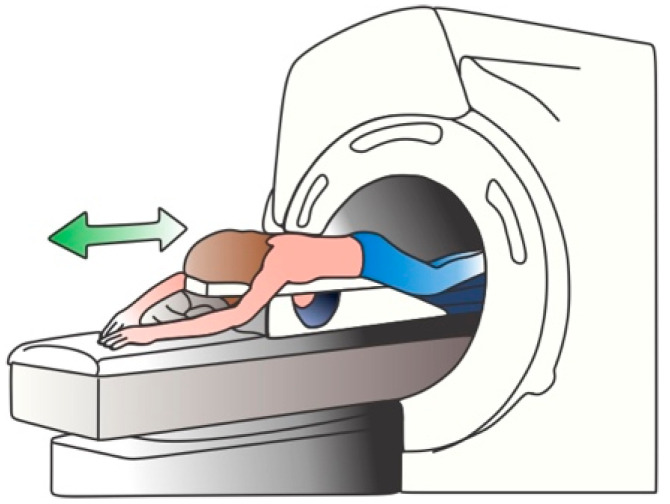
BMRI procedure.

Breast MRI (BMRI) uses a magnetic field and radio waves to create a detailed image from the breast. Usually, a 1.5 T magnet is used along with a contrast, usually gadolinium, to generate the images of both breasts [22]. To acquire the images, the patient is located in a prone position, in order to minimize the respiration movement and to allow the expansion of the breast tissue [10,22]. When the magnet is turned on, the magnetic field temporary realigns the water molecules; thus, when radio waves are applied, the emitted radiation is captured using specific-designed coils, located at the breast positions, which transforms the captured radiation in electrical signals. The coils position must ensure an appropriate field-of-vision from the clavicle to the infra-mammary fold, including axilla [10]. An illustration of the patient position is depicted in Figure 4.

The main objective of getting the images is to assess for the breast symmetry and the possible changes in the parenchymal tissue, since those changes might indicate the presence of lesions that can be malignant. In general, malignant lesions have irregular margins (or asymmetry), whereas the benign ones usually have a round or oval geometrical shape with well-defined margins (symmetry). To deliver the best possible result, it is necessary to remove the homogenous fat around the breast and parenchyma since fat can render images that can be uninterpretable, specially to detect subtle lesions [10,22].

On the other hand, one of the problems that BMRI has is the false-positive (specificity) rate, as the technique can detect low-size masses (lesions whose size is less than 5 mm) that are benign [10,22]. To mitigate the aforementioned issue, nanomaterials have been developed, so they stick to the cancer masses but not to the benign ones [23] as well as contrast agents [24]. Recently, it has been proposed that a multiparametric approach has been suggested as a strategy to improve the specificity rate [10].

### 2.4. Other Approaches

Recently, microwave radiation has been employed as an alternative to obtain information about the breast tissue. The microwaves, whose frequency range varies from 1 to 20 GHz, are applied to the breast and the reflected waves are measured using specific-designed antennas. To have the best possible results, some works propose that the tissue must be immersed in a liquid [25]. In this sense, some works have proposed acquisition systems that deal with this issue [26,27,28,29].

When it is necessary to perform a biopsy to confirm, images from the cells that form the abnormalities are obtained using among other techniques, the fine needle aspiration citology (FNAC), core or excisional biopsy. Once the cell images are captured, an image processing technique is applied in order to detect the differences between normal and malignant cells, which are classified using modern strategies [30,31,32] such as neural networks, probabilistic-based algorithms and association rules coupled with neural networks.

It should be pointed out that other alternatives for imaging are employed such as Computed Tomography (CT) or Positron Emission Tomography (PET). The former employ X-rays to form images from the chest using different angles; using image processing and reconstruction algorithms, a 3D image of the chest (including the breasts) is obtained [33,34]; on the other hand, the latter uses a small amount of tracer, that is a specific-designed sugar with radioactive properties known as fluorodeoxyglucose-18. The main idea of using this type of sugar is that cancer cells have an increased consume of glucose compared with the normal cells; in this sense, the tracer sticks in the zones where there is an increased glucose consume [35,36]. It is worth noticing that these techniques are recommended to determine the cancer stage rather than first-line diagnosis scheme [10,37]. In this way, they complement the three main techniques to provide more information from the tissues surrounding the breasts [37]. Table 1 presents a table that summarizes the abovementioned methods.

As it is seen in Table 1, numerous advances for imagining techniques have been achieved in the last years; still, there is a necessity of developing strategies that can allow obtaining sharp images, even for dense breast tissues. In this sense, the obtained images can be used to perform a focused surveillance on the patients that have a higher risk for developing the disease, allowing to achieve the cancer detection in the earliest possible stage. On the other hand, these novel imagining techniques should be able to operate without requiring additional requirements, such as specific electrical or mechanical conditions, so they can be easily adopted in hospitals, or in an ambulatory area.

## 3. Image Processing and Classification Strategies

### 3.1. ROI Estimation

Once the image is acquired, the next step required is its interpretation. To this purpose, it is necessary to identify the suspicious regions that might contain masses or calcifications, where model, region, or counter-based algorithms for the image segmentation are employed [45]. It should be noticed that these approaches often rely on the manual entries to refine the segmentation zones, which limits the applicability of the proposals on different datasets [45], making necessary to develop novel strategies that can automatically detect all the interest zones. Recently, Sha et al. [46] proposed a convolutional neural network (CNN)-based method for segmentation. The authors develop an optimization scheme to determine the best parameters for the CNN in order to segment the suspicious zones. The results presented show the proposal has a reasonable sensitivity and specificity (89% and 88%, respectively) to determine if a mammograph presents cancerous tumors or not. Wang et al. [47] present a CNN-based strategy. They modify the convolutional layer to increase the detection of multiple suspicious zones. Heidari et al. [48] employ a Gaussian bandpass filter to detect suspicious zones using local properties of the image. On the other hand, Suresh et al. [49] and Sapate et al. [50] employ a fuzzy-based strategy to cluster all the pixels with similar features in order to detect all the zones that have differences. Other strategies involve the utilization of mathematical morphology [51,52,53,54,55], image contrast and intensity [56,57], geometrical features [58,59], correlation and convolution [60,61], non-linear filtering [62,63], texture features [64], deep learning [65,66,67,68,69], entropy [70,71], among other strategies. It is worth noticing that from the diversity of the employed strategies, some of them still require an initial guidance to detect the suspicious zones, either by manually selecting pixels inside of the zone or using the radiologist notes about the localization. An effective approach for the automatic detection should employ a denoising stage in order to remove residual noise generated during the acquisition and equalization, so the intensity pixel disparities associated to the environment light can be mitigated as much as possible.

### 3.2. Feature Extraction

After the suspicious zones are detected and segmented, it is necessary to extract features from them to generate the necessary information to classify the detected lesions as cancer or benign. To this purpose, Fourier Transform-based methods [48,72], wavelet transform-based strategies [73,74,75,76], geometric features [77,78], information theory algorithms [79], co-occurrence matrix features [47,80,81,82], histogram-based values [46,83,84,85], morphology [86,87], among others. On the other hand, with the increased capabilities (the number of simultaneous operations that can be done) of the new-generation graphical processor units, it is now possible to execute high-load computational algorithms faster than in a multicore processor [88]; in consequence, novel neural networks algorithms that perform the feature extraction and quantification are now being proposed. For instance, Xu et al. [89], use a CNN to extract and classify ultrasound images with suspicious areas in four categories: skin, glandular tissue, masses, and fat. They modify the convolutional filters to speed up the process. Arora et al. [90] also use an ensemble of CNN architectures to extract directly the suspicious zones. They only modify the final layers to speed up the training process. Gao et al. [91] use a deep neural network to generate the features from mammograms. They employ a modified architecture where the outputs and inputs of the network are used to update the model parameters during its training. Similar approaches are described in [92,93,94,95].

It should be pointed out that a reduction of the estimated features is often used to reduce the amount of computational resources used in the training scheme and to mitigate the overfitting problem, which reduce the algorithm efficacy. This step is known as dimensionality reduction [45] and the most employed algorithms are the principal component analysis (PCA) and linear discriminant analysis (LDA). PCA use eigenvalue-based algorithms to determine the features that are unrelated between them, that is, they have the maximum variance between them as this will indicate the maximum variation of the information contained, whereas LDA perform a projection of the samples to find out the distance between the classes’ mean. In this sense, the greater the distance between the means, the more unrelated the features are [96]. Nevertheless, these algorithms use global properties of the values which might cause to deliver suboptimal results [96]. For these reasons, hybrid strategies are proposed such as neurofuzzy algorithms [97,98], diffusion maps [99], deep learning [100,101,102], independent component analysis (ICA) [103], clustering-based approaches [104], multidimensional scaling [105], among other strategies. It should be pointed out that hybrid approaches, as abovementioned ones, are particularly effective when a non-linear relationship between the features exists.

To the best of the authors’ knowledge, there are no papers that compare some of the abovementioned techniques using the same database to compare the techniques efficacy. In this sense, it is an interesting research topic, since the results of this comparison can provide some guidelines about the image used (mammogram, ultrasound, or MRI) and the technique that has the best performance.

### 3.3. Classifiers

The last step of this stage is the classification of the extracted features to make a diagnosis. Broadly speaking, a classifier uses the input data to find out relationships that can be used to determine the class where the input data belongs to. The evaluation of the classifier is done using three basic measurements: accuracy, specificity, and sensitivity [106,107]. Accuracy refers to the percentage of images that are correctly classified in their corresponding classes; sensitivity is the percentage of classified images as malignant that truly are specificity is the percentage of classified images as benign that truly are, and the area under the curve is a parameter that allows choosing the optimal models. It takes a value between 0 and 1, being a good classifier the one that has a value close to 1 [108]. In this sense, depending on the training algorithm required by the strategy, classifiers can be divided in unsupervised or supervised [45,106,107].

#### 3.3.1. Unsupervised Classifiers

An unsupervised classifier aims to find the underlying structures that the input data has without making explicit the class the input data belongs to [109]. In this sense, input data that has similar values is assigned to the same class [109]. Dubey et al. [110] studied the effects that the selection scheme for the size of the number of clusters in the K-means algorithm has. To this purpose, the random and foggy methods were employed. They note that foggy initialization method and the Euclidean-type distances produced the best results, as a 92%-accuracy is obtained. K-means and K-nearest neighbor classifiers have been also employed by Singh et al. [58] and Hernandez-Capistran et al. [111]. This family of classifiers is effective when the distance between the clusters is reasonable; but, when the aforementioned concept is not possible, the accuracy rate is highly degraded. For this reason, Onan [112] introduced the concepts of the fuzzy logic to measure the distance between the set of features used as input and the clusters, where the mutual information, an information theory algorithm, is the chosen to measure the aforementioned distance. The author reports an accuracy of 99%, and a specificity and sensitivity of 99% and 100%, respectively. Similar results are achieved using the fuzzy c-means algorithm [113,114], fuzzy-based classifier for time-series [115], fuzzy rule classifier [116,117], among others. Other clustering-based approaches employed for classification are hierarchical clustering [118] and Unsupervised Test Vector Optimization [119]. It should be pointed out that unsupervised classifiers require a careful selection of the features used to train the algorithm, since an incorrect mix of features will degrade the performance of the classifier.

#### 3.3.2. Supervised Classifiers

Supervised classifiers require to know a-priori the class of which the input data belongs to, that is, the input data must be labeled. The Decision Tree (DT) is an algorithm that uses a set of rules to determine the class of the data input. DT has been employed by Mughal et al. [71], where they perform the detection of masses in mammograms using texture features in the region of interest. Using a DT, they obtain an accuracy, specificity, and sensibility of 89%, 89% and 88.5%, respectively. Shan et al. [120] employ geometrical features to classify abnormalities detected in ultrasound images. The obtained results show an accuracy, sensitivity, and specificity of 77.7%, 74.0%, and 82.0%, respectively. An improvement of DT is the Random Forest (RF). During the training stage, RF uses several DT, where the ones that have the lowest error are chosen; in this way, the accuracy is enhanced. RF are considered as ensemble classifiers, where some applications have been reported [121,122,123,124]. The accuracy, specificity, and sensitivity reported show an improvement. Another type of ensemble classifier is the Adaptive Boosting (AdaBoost) algorithm. It consists in the utilization of weak classifiers, which are usually features that can generate a classification accuracy greater than 50% by themselves; thus, using them in an ensemble way, the outliers that the features value have are used, improving the classifier accuracy. AdaBoost applications have been reported [125,126,127], achieving good results (the accuracy, specificity, and sensitivity values are greater than 90%); yet, the authors note that extensive investigation is still required to ensure that these results can be obtained with different types of images (mammograms, ultrasound, and MRI).

Another classification algorithm widely used for BC detection is the support vector machine (SVM). SVM finds the hyperplane that divides the zones where the values of the input features are located. In this regard, Liu et al. [52] use the morphological and edge features combined with a SVM classifier with a linear kernel, to detect benign and malignant masses in ultrasound images. They obtain an accuracy, sensitivity, and specificity of 82.6%, 66.67%, and 93.55%, respectively. It should be noted that most of the revised works use the term malignant to describe masses or lesions that are cancer regardless its type. To improve the aforementioned results, Sharma and Khanna [128] use the Zernike moments as features and a SVM classifier using a non-linear function as a kernel. The authors obtain a specificity and sensitivity of 99%. Similar approaches have been reported [87,129,130,131,132,133]. It is worth noticing that if the features have a strong nonlinear relationship, other classifiers could deliver better results.

### 3.4. Artificial Intelligence-Based Classifiers

**Figure 5:**
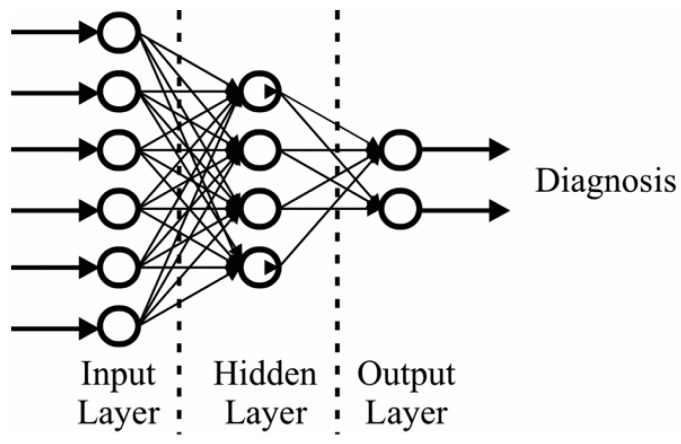
Artificial Neural Network.

Artificial Intelligence (AI) is the section of the computer science that develops algorithms to perform complex tasks that previously are solved with the human knowledge [134]. Evidently, since classification is a task usually solved by the physician, AI can provide automated solutions. In this sense, Artificial Neural Networks (ANN) are a type of AI algorithms employed to perform the classification in different classes. ANN are brain-inspired algorithms that store the knowledge that the input data using a training process [135]. An ANN consists in a three-layer scheme: input, hidden, and output, as depicted in Figure 5.

The training process takes the information contained in the input variables and adjust the values of the variables (weights) that connect all the layers in order to match the input with its respecting class; in this way, the hidden pattern that share all the input and their corresponding class is detected and stored. Consequently, it is necessary to use a sufficient database, with representative scenarios, to train the ANN. Beura et al. [136] present a methodology that employs mammograms to detect masses (benign and malignant) using the two-dimension discrete wavelet transform (2D-DWT) with normalized gray-level co-occurrence matrices (NGLCM). The images are segmented using a cropping-based strategy to obtain the ROI, which are analyzed with the symmetric biorthogonal 4.4 wavelet mother and a decomposition level of 2. All the frequency bands are processed to obtain the features (NGLCM), where the *t*-test is selected to perform the optimal choice of the most discriminant features. The obtained results show that the proposal achieves an accuracy, sensitivity, and specificity of 94.2%, 100%, and 90% respectively, using the ANN classifier, whereas a RF classifier, using the same database, obtains an 82.4%-accuracy. Mohammed et al. [137] uses fractal dimension values as features to classify ultrasound breast images in benign and malignant. They obtain the ROIs using a cropping-based algorithm, which are processed to obtain multifractal dimension features. They obtain an accuracy, sensitivity, and specificity of 82.04%, 79.4%, and 84.76% respectively using an ANN classifier. They point out that the ROI extraction algorithm must be improved. Gallego-Ortiz and Martel [138] classifies MRI breast images using graph-based features, the Deep Embedded Clustering algorithm to select the most relevant features and an ANN classifier. The ROIs are obtained using a graph model, where they obtain an area under the curve, which is another feature to measure the classifier effectiveness, of 0.80 (the closer to 1, the better). ANN classifiers have been also used in [139,140,141,142].

**Figure 6:**
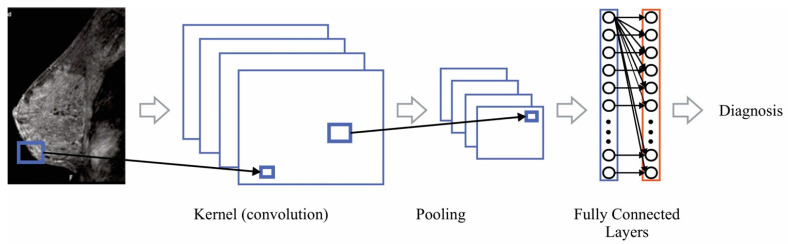
Convolutional Neural Network.

Deep neural networks (DNN) are a specific type of AI algorithms based on the architecture of an ANN [134]. DNN resembles how the brain stores, in multiple layers, the acquired knowledge to solve a specific task [8]. The Convolutional Neural Network (CNN) is a DNN that emulates the visual processing cortex to determine the class that an image belongs to [8,134]. A CNN typical scheme is depicted in Figure 6.

From the figure, it is seen that a CNN consists of a kernel, pooling and fully connected layers. The purpose of the kernel layer is to detect and extract spatial features that the image has, which is usually done with the convolution operator. The output of this layer, known as feature map, might contain negative values that might cause numerical instabilities in the training stage; thus, map is processed using a function to avoid the negative values. Once the feature map is processed, the pooling layer reduces the amount of information contained in order to eliminate redundant information; finally, the output of the pooling layer goes to the fully connected layer to be classified. In this sense, several works [143,144,145,146,147,148], have been employed CNN to detect benign and malignant tissues in either mammography or MRI images. They note that the depth of the network, i.e., the number of layers, the fine-tuning of some of the kernel or pooling layers, as well as the number of images, affect the classifier performance.

Ribli et al. [149] add an additional layer to implement specific-designed filters for mammograms. The CNN they employ has 16-layers and classifies the detected lesions in benign or malignant, obtaining an area under the curve of 0.85. A similar approach is proposed in [150]. The modification they propose is that a fully connected layer is placed as the first layer of the CNN so when the images are noise-corrupted, the feature extraction process is not degraded. They obtain an accuracy, sensitivity, and specificity of 98.7%, 98.65%, and 99.57% for the detection of benign and malignant lesions in mammograms. Zhang et al. [151] carry out a test to find out the specific-suited process for the pooling layer. They found out that rank-based stochastic process is the best-suited algorithm, obtaining an accuracy, sensibility, and specificity of 94.0%, 93.4%, and 94.6%, respectively, for classifying lesions for normal or abnormal using mammograms. Similar approaches have been proposed [152,153,154,155]. Table 2 presents a summary of the classifiers above discussed. It should be noted that a mix of images from mammograms, ultrasound, MRI are usually employed. These images usually came from private databases.

From the data shown in Table 2, it can be seen that it is necessary to standardize the minimum requirements regarding the number of images that the databases must have. In this way, the performance metrics that are employed, i.e., accuracy, specificity, and sensitivity, can be compared in a better way. Moreover, even when the presented approaches show interesting results, one thing they found out is the necessity of having a considerable database that contain significant labeled images to obtain the best possible results, which in many real-life scenarios is not always possible. For these reasons, algorithms that can work with both labeled and unlabeled images are still a necessity.

## 4. Recent Image Generation Techniques

### Infrared Thermography (IRT) Applied to Breast Cancer

**Figure 7:**
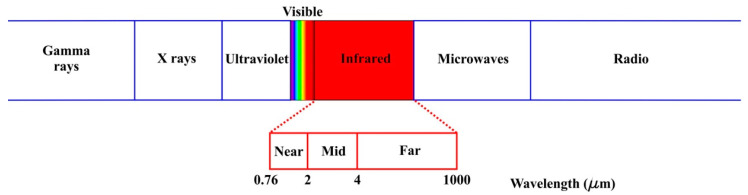
Electromagnetic spectrum.

Temperature has been documented as an indicator of health [156]. Specifically speaking of breast cancer, when a tumor exists, it makes use of nutrients for its growth (angiogenesis), resulting in an increase in metabolism, thus the temperature around the tumor will increase in all directions [157]. To detect the temperature changes, IRT has been used as it measures the intensity of the thermal radiation (in the form of energy) that bodies emit, converting it into temperature [158]. The emitted energy can be visualized in the electromagnetic spectrum, as shown in Figure 7, where it is seen that the infrared (IR) wave ranges from 0.76 to 1000 μm and in turn is divided into Near-IR, Mid-IR and Far-IR. The available technology to measure IR allows performing the aforementioned task using non-invasive, contactless, safe, and painless equipment [159,160,161], making a suitable proposal for developing scanning technologies.

To obtain the best possible images, there are mainly three factors that influence thermographic imaging in humans [162,163]

1. Individual factors: everything that has to do with the patient’s conditions, such as age, sex, height, medical history, among others. As well as the inclusion and exclusion criteria. An aspect of vital importance is the emissivity of humans, which is 0.98 [164].
2. Technical factors: it has to do with everything related to the technology used during the study, such as the thermal imager (considering the distance from the lens to the patient), the protocol, the processing of the medical thermal images obtained, as well such as feature extraction and subsequent analysis.
3. Environmental factors: room position (it should be located in the area of the lowest possible incidence of light), temperature, relative humidity of the space where the thermographic images are to be taken, as well as the patient’s air conditioning time.

**Figure 8:**
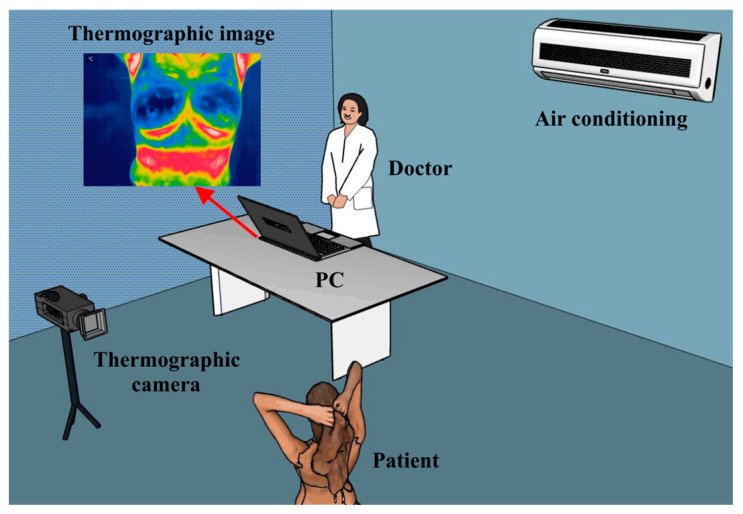
Proposed experimental set up for the breast thermal images acquisition.

Considering the all the above discussed aspects, a suitable location for developing a controlled scenario to acquire thermographic images focused on breast cancer is depicted in Figure 8.

Once the room is conditioned for obtaining the thermographic images, the acquisition can be done. The reported results make use of the previously discussed image processing and classification algorithms. Table 3 shows a brief resume of the most recent proposed works.

Recently, dynamic infrared thermography (DIT) has been proposed as an alternative to further improve the image quality and sharpness [64]. DIT is a sequence of thermograms captured after stimulating the breasts by means of a cold stressor [176]. The objective of this stressor is to generate a contrast between areas with abnormal vascularity and metabolic activity with areas free of abnormalities. Therefore, it is possible to analyze the sinus response after removing this stimulus. In this way, the image sharpness is enhanced. Silva et al. [177] proposed a technology that analyzes the information from the DIT to indicate patients at risk of breast cancer, where they segment the area of interest (breast) and analyze the changes in temperature through the different thermograms acquired. Saniei et al. [178] proposed a system that segments both breasts to obtain the branching point of the vascular network, which represents the pattern of the veins; finally, these patterns are classified to obtain the diagnosis. As it can be seen, the DIT requires robust systems that allow the analysis of the acquired thermograms over time, which should be considered in order to generate the next generation of equipment that can allow the early detection of the angiogenesis process. By doing this, patients can be properly monitored so the changes in the patterns of the angiogenesis process be detected.

## 5. Recent Classification Algorithms

As pointed out in the Classifiers subsection, it is necessary to overcome the lack of a large database of images (mammograms, ultrasound or BRMI) that have been diagnosed to generate robust and efficient classifiers. In this sense, semi-supervised methods can be an attractive choice to explore. They usually combine an unsupervised algorithm to cluster the images available, so a representation of the dataset is obtained; then, the supervised classifier assigns the classes that images have [109,179]. The data that is used in the unsupervised algorithm assumes the unlabeled images are close to the labeled ones in their input space, so their labels are the same [109]. Some of the most recent developments that could be applied in the breast cancer detection are presented.

### 5.1. Autoencoders

**Figure 9:**
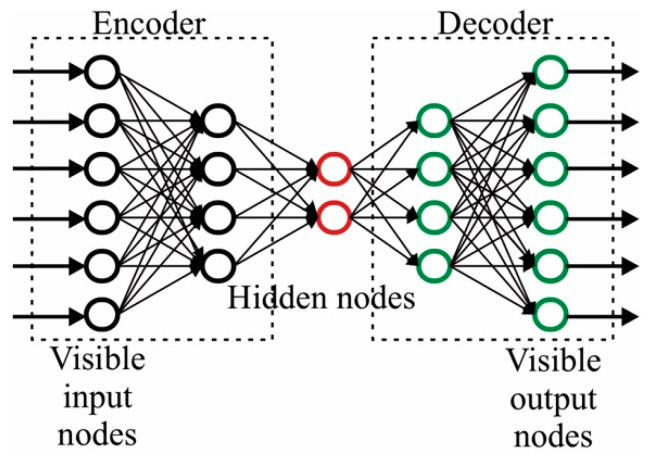
Autoencoder structure.

An autoencoder is a neural network that has one or more hidden layers that is used to reconstruct the input compactly, as the hidden layers have few neurons. The autoencoder is depicted in Figure 9.

From the figure, it is seen that it has two parts: the encoder, that represents the input into its compact representation, and the decoder, which performs the inverse operation, that is, use the compact representation to recover the original data. The most common training scheme consists in employing a loss function that aims to reduce the error between the original and reconstructed data. For breast cancer detection, autoencoders can be used feature extraction stages, as the encoder part obtains the compact representation or features of the input image, that are followed by a supervised classifier. Recently, this approach has been explored [79,94,180,181,182,183] showing promising results to generate robust methodologies, where accuracies values above 95% are obtained.

### 5.2. Deep Belief Networks (DBF)

They are based on the usage of restricted Boltzmann machines (RBMs). RBMs only use two layers: input and hidden, to represent, as in the case of the autoencoders, the most important features that can represent the input data but in a stochastic way [99]. This ensure that the outliers do not affect the network performance. Detailed information can be found in [184,185]. The main idea in employing DBF is that the image segmentation can be done without external guidance; thus, a totally automated methodology can be proposed. Recent works have been explored this idea to perform the liver segmentation [186], lung lesions detection [187], and fusion of medical images [188]. Its use could deliver promising results to detect BC.

### 5.3. Ladder Networks

Ladder Neural Network, proposed by Rasmus et al. [189], uses an autoencoder as the first part of a feedforward network to denoise the inputs; further, by determining the minimum features that represent the inputs, the classification can be done using simple algorithms. The network uses a penalization term in the training algorithm to ensure the maximum similarity between the original and reconstructed inputs.

### 5.4. Deep Neural Network (DNN)-Based Algorithms

Recently, DNN-based classification strategies have been proposed to maximize the accuracy that the classifiers achieve while reducing the computational resources required to perform its training and execution, being the physics-informed neural network or more recently, the Deep Kronecker neural network [190] are one of the most recent algorithms that have been proposed. In particular, these NNs are designed to take full advantage of the adaptive activation functions. Traditional activation functions, such as the unipolar and bipolar sigmoid and the ReLU, might have problem when dealing with low-amplitude features as the training algorithm fails to achieve the lowest point in the error surface, thus generating classifiers prone to have generalization issues [190].

In this sense, by introducing a parameter into the activation function equations that can be modified during the training process, it can be avoided that the gradient function does not stall in a local minimum on the error surface [191]; thus, the highest accuracy can be obtained since the global minimum is reached [192]. The results presented [190,191,192] suggest that the utilization of this type of activation function might increase the classifier accuracy without increasing the computational burden required to train the network as the geometrical shape that the activation function defines can be adapted during the training time to the boundary decision zone where classification is required. It should be noted that the proposed Rowdy family of activation functions could be an interesting research topic for designing classification algorithms, as the presented results demonstrate that the lowest error is achieved in a prediction task.

## 6. Concluding Remarks

This paper presents a state-of-the-art review of the technologies used to acquire images from the breast and the algorithms used to detect BC. To the best of the author’s knowledge, this is the first review article that deals with all the required steps to propose a reliable methodology for the BC detection. This is important as the earliest detection of the disease can save a considerable amount of money in the required treatments, and the most important, potentially saving numerous lives.

The analyzed papers are focused on the research on the processing of images obtained using non-invasive methods: X-ray, ultrasound, or magnetic resonance, as they are the most accessible technologies in hospitals. The strategy used in most of the papers has 4 steps: image acquisition, ROI estimation, feature extraction, and interpretation. For the ROI estimation, the strategies proposed are based on radiologist annotations or require external help in order to be executed. This is an opportunity area to develop automatic algorithms that can detect the abnormalities. The feature estimation is used to quantify the detected zones in numerical values. In this sense, texture-based and geometrical-based features are by far, the most employed due to its estimation simplicity; still, frequency or spatial features have recently begun to be explored and can lead to detect minimal changes that might increase the sensitivity required to further improve the classification accuracy. It should be noticed that feature reduction strategies are commonly employed in order to reduce the training time or avoid potential misclassifications, where the most popular are LDA and PCA. On the other hand, classification strategies employ either supervised or unsupervised algorithms. The selection of the type of classifier heavily depends on the nature of the features extracted. If they are highly discriminant between them, then an unsupervised classifier is usually selected. On the other hand, when the features used have an overlap zone, then it is necessary to employ a supervised classifier. It should be noticed that AI-based algorithms, especially those based on deep learning, have the edge in terms of the performance they get at the expense of being very expensive in terms of the computational resources employed.

Emerging imaging technologies such as the microwave and thermography are being explored recently. In particular, the latter has recently obtained the attention of researchers as it is easy-to-use, and, with a proper cooling protocol, can reach an interesting level of accuracy to detect, at least, suspected masses that might evolved into malignant ones. With the development of semi-supervised strategies, some of the stages employed can be integrated into one, allowing the development of effective feature extraction, selection and classification strategies that have the same performance of supervised classifier, with lower computational resources employed, even in the presence of limited labeled images, which is a major obstacle to the training of the classifiers.

Modern BC detection strategies should rely using artificial intelligence(AI)-based algorithms that can use both on the information of the images acquired and categorical data [193,194,195], i.e., information about the daily life of the patients, with the aim of proposing algorithms that can determine if the patient has malignant lesions with a higher certainty and with the lowest false alarm at the earliest stage possible in order to get an effective treatment that can prevent the disease propagation. To achieve this goal, it is necessary to develop a database that contains the aforementioned features and whose size can reflect the main scenarios that can be found in real-life. Further, having algorithms that can deal with the aforementioned information, it can be possible to design personalized surveillance and clinical screening strategies that could offer the best health outcome for every patient.

## Figures and Tables

**Table 1:** Summary of the used breast image generation technologies.

Imagining Technique	Advantages	Disadvantages	Recommended Population	Some Types of Cancer Detected	Sensitivity and/or Specificity
Mammography	1. Equipment is widely available worldwide. 2. Methods, such as tomosynthesis, can improve the specificity and sensibility of the technique with patients that have dense breasts [10]	1. The rate of both false positive and false negatives increases since there is no possibility to determine if the masses are benign 2. The procedure used to obtain the images could be bothersome. 3. Dense breasts or young patients are not indicated to use this imaging technique.	Women whose age is greater than 40 years, have low-dense breast and an average risk of contracting the disease.	1. Ductal Carcinoma in Situ 2. Invasive Breast Cancer.	Sensitivity up to 85%.
Ultrasound	1. Can be used in young patients or have dense breast. 2. The equipment used is available in most of the hospitals	1. Calcifications could not be detected. 2. Sensitivity depends on the operator ability to interpret the images 3. False-positivity rate is an issue.	Women with heterogeneously or extremely dense breast tissue [38,39]. Women that are pregnant or lactating [40].	1. Ductal Carcinoma in Situ. 2. Invasive ductal carcinoma	Sensitivity ranging between 40–75% in younger high-risk women [40].
Magnetic Resonance Imaging	1. Effective for detecting suspicious masses in high-risk population [10]. 2. The breast tissue density is no longer an issue [38,39,40]. 3. Multifocal lesions can be detected [10,41]	1. Equipment is only available in specialized hospitals. 2. Expensive 3. False positive findings are an important concern [41]	1. Women that may carry mutation in ATM, BRCA1, BRCA2, CHEK2, PALB2, PTEN, TP53 genes. 2. Women that had radiation therapy in the chest zone during the childhood.	1. Ductal in situ carcinomas 2. Invasive ductal carcinomas. 3. Invasive lobular carcinomas 4. Invasive mammary carcinomas with mixed ductal and lobular features [24]	Sensitivity ranging from 83 to 100% [42,43,44].

**Table 2:** Summary of the used image classification algorithms.

Type of Classifier	Classifier	Advantages	Disadvantages	Number of Images	Performance Metrics
*Unsupervised*	K-means	Easiness of implementation. Fast implementation. Fast computing (distance to the centroids is only required).	The initial value of the centroids length influences the performance. Samples must be presented in an organized and normalized way. The centroid distance of the classes might induce misclassifications.	Some works have used the Wisconsin Breast Cancer Dataset with 569 instances [110,112].	Accuracy: up to 92% [110] Specificity: up to 99% [112] Sensitivity: up to 100% [112]
	Hierarchical Clustering	No distance measurement is required. Similarity measures could be employed. Easy to implement.	Large datasets increase the time complexity to deliver a result. Outliers degrade the classifier performance. Normalization of the samples values is required.	117 images are analyzed [118].	Accuracy: 88.0% Specificity: 89.3% Sensitivity: 85.7% [118]
*Supervised*	Decision Trees	Its construction no imposes any probabilistic distribution to the data. Can deal with large datasets. Easy to understand.	They can be too complex if the training data is not carefully chosen. Their performance will decrease if several classes exist in the data.	Some works have analyzed from 283 [120] to 722 images [71].	Accuracy: up to 89%, Specificity: up to 89% Sensitivity: up to 90% [71,120]
	Random Forest	Non-linear relationships between the features are well processed. Outliers do not degrade the classifier performance. Noisy measurements do not affect the accuracy.	Training time increases due to the number of trees generated. The classifier complexity is increased as the number of trees needed to be evaluated.	Several works have used different number of images from 59 [121], 283 [120] to 512 [122]. On the other hand, some authors have used ten different datasets, the shortest with 155 images and the largest with 569 images [123].	Accuracy: up to 80%. Specificity: up to 80%. Sensitivity: up to 90% [120,121,122,123]
	AdaBoost	Base classifiers only need to have an accuracy greater than 50%. They can be from different domains (spatial, frequency, among others)	Noise can degrade the classifier performance, as the weight assigned to each weakly classifier is increased to reduce the error. Sensitive to the base classifiers employed.	Some works have used from 1062 [126] to 2336 [125] images.	Accuracy: up to 90%. Specificity: up to 90%. Sensitivity: up to 90% [12,125,126]
	Support Vector Machines	Can deal with high-dimensional data (features). Robust against outliers. Overfitting is reduced due to the training process.	Accuracy is kernel dependent. Large datasets are not properly handled. Overlapping and noise degrade the accuracy. Uncertainty cannot be incorporated.	Some authors have used different number of images from 207 [87], 240 [132] to 1187 [131].	Accuracy: up to 90%. Specificity: up to 90%. Sensitivity: up to 90% [74,87,131,132]
	Artificial Neural Networks	Can deal with highly non-linear relationships. Can deal with noisy data. Uncertainty can be incorporated. Fine-tuning could be done using different activation functions.	High-dimensional data might cause instabilities to the training algorithms. Prone to overfitting. Selection of the number of neurons could be troublesome.	Other authors have been used 111 [139], 184 [137], and 569 [140] images.	Accuracy: up to 95%. Sensitivity: up to 100%. Specificity: up to 90% [134,137,138,139,140]
	Convolutional Neural Networks	Can process the image without any preprocessing stage. They can perform feature extraction task automatically. Moderate noisy images can be properly handled.	They require a large dataset to avoid overfitting. They require a high computational load to their training.	Some authors have used different number of images from 87 [144], 221 [143] to 229,426 digital screening mammography exams [145].	Accuracy: up to 99%. Sensitivity: up to 99% Specificity: up to 99.6% [7,8,143,144,145,149]

**Table 3:** Summary of the breast lesions detection using infrared thermography.

Authors	Number of Patients	IR System	Image Processing and Classification Algorithms		Accuracy (%)	Room Temperature (°C)	Acclimation Time (min)
			Features	Classification			
Ekici and Jawzal [165]	140	FLIR SC-620	Bio-data, image analysis, and image statistics	CNNs optimized by Bayes algorithm	98.95	17–24	15
AlFayez et al. [166]	Public dataset DMR-IR		Geometrical and textural features	Extreme Learning Machine (ELM) and Multilayer Perceptron (MLP)	ELM—100 MLP—82.2	Public dataset DMR-IR	
Rani et al. [167]	60	FLIR T650SC	Temperature and intensity	SVM with Radial basis function kernel	83.22	20–24	15
Saxena et al. [168]	32	FLIR A320	ROI thermal	Cut-off value	88	22 ± 0.5	Not specified
Tello-Mijares [169]	63	FLIR SC-620	Shape, colour, texture, and left and right breast relation	CNN	100	20–22	15
Garduño-Ramón et al. [170]	454	FLIR A300	Temperature	Difference of temperature	79.60	18–22	15
Raghavendra et al. [171]	50	Thermo TVS200	Student’s *t*-test based feature selection algorithm	Decision Tree	98	20–22	15
Lashkari et al. [172]	67	Thermoteknix VisIR 640	23 features, including statistical, morphological, frequency domain, histogram and Gray Level Co-occurrence Matrix	Adaboost, SVM, kNN, Naive, PNN	85.33 and 87.42	18–23	ice test: 20 min
Francis et al. [173]	22	med2000™ IRIS	Statistical and texture features are extracted from thermograms in the curvelet domain	SVM	90.91	25	15
Milosevic et al. [174]	40 images	VARIOSCAN 3021 ST	Texture measures derived from the Gray Level Co-occurrence Matrix	K-Nearest Neighbor	92.5	20–23	Few minutes
Araujo et al. [175]	50	FLIR S45	Thermal interval for each breast	Linear discriminant classifier, minimum distance classifier, and Parzen window	-	24–28	At least 10 min

## Data Availability

Not applicable.
